# Digestive Tract Cancer: A Retrospective Study of the Epidemiological Profile of Populations Exposed and Not Exposed to Uranium Contamination in North Kazakhstan (2014–2023)

**DOI:** 10.3390/jcm15114330

**Published:** 2026-06-03

**Authors:** Kuralay Ilbekova, Danara Ibrayeva, Elena Saifulina, Mulkat Yelshenbek, Moldir Aumalikova, Kairullova Madina, Dinara Bizhanova, Gulnur Doszhanova, Bekenova Farida, Yerbol Dogalbayev, Meirat Bakhtin, Polat Kazymbet

**Affiliations:** 1 Scientific Research Institute of Radiobiology and Radiation Protection, NJSC Astana Medical University, Astana 010000, Kazakhstansaifulina.e@amu.kz (E.S.); yelshenbek.m@amu.kz (M.Y.); aumalikova.m@amu.kz (M.A.); kairullova.m@amu.kz (K.M.); bizhanova.d@amu.kz (D.B.); bakhtin.m@amu.kz (M.B.); p.kazymbet@mail.ru (P.K.); 2 Scientific Research Institute of Preventive Medicine Named after Dalenov E, NJSC Astana Medical University, Astana 010000, Kazakhstan; doszhanova.g@amu.kz; 3 Department of Internal Medicine, NJSC Astana Medical University, Astana 010000, Kazakhstan; bekenova.f@amu.kz; 4 Department of Interventional Radiology, CF University Medical Center, Astana 010000, Kazakhstan

**Keywords:** digestive tract cancers, uranium waste storage site, cancer incidence, relative risk, North Kazakhstan, retrospective registry study

## Abstract

**Background/Objectives**: Cancer incidence and mortality are key epidemiological indicators for evaluating the long-term biological effects of chronic low-dose radiation exposure. The aim of this study was to assess the incidence, structure, and relative risk of digestive tract cancers (ICD-10 C15–C26) among populations exposed and not exposed from uranium waste storage site in North Kazakhstan over a 10-year period (2014–2023). **Methods**: A retrospective population-based analysis was conducted using national cancer registry data, including data for residents of Stepnogorsk and nearby settlements (exposed group) and Akkol (control group). **Results**: A total of 588 cases were identified, including 465 in the exposed group and 123 in the control group. The mean age at diagnosis was similar (~65 years), and most cases were diagnosed at advanced stages (III–IV: ~58–60%). The leading cancer types were colorectal (38–40%) and stomach (29–31%) in both groups. The incidence rates were comparable (85.27 vs. 87.40 per 100,000), with an overall relative risk (RR) of 0.98 (95% confidence interval [CI]: 0.80–1.19), indicating no significant difference. Site-specific analysis showed no significant variation for esophageal, stomach, or colorectal cancers. A higher, but not statistically significant, risk was observed for pancreatic cancer (RR = 1.61; 95% CI: 0.90–2.89). Temporal analysis demonstrated similar trends in both populations, with incidence rates decreasing from 91.37 to 79.08 per 100,000 in the exposed group and from 97.40 to 78.13 per 100,000 in the control group between 2014–2018 and 2019–2023. **Conclusions**: Overall, digestive tract cancer incidence and structure were comparable between groups, with no statistically significant increase in the exposed population.

## 1. Introduction

Kazakhstan is one of the world’s leading countries in terms of uranium reserves and production, with six major uranium ore provinces. These regions contain numerous uranium mining and processing sites that, while economically important, may also contribute to long-term environmental and radiological burdens. In many instances, these facilities are located near residential settlements, creating conditions for chronic population exposure to radiation-related factors. Consequently, contamination of environmental areas, including air, soil, water, and food products, has become an important issue for public health and environmental safety [1,2].

For this study, uranium waste storage site (UWSS) refers to uranium-related environmental contamination associated with former uranium mining and processing activities, including uranium tailings and uranium waste storage sites. Digestive tract cancers were defined as malignant neoplasms classified under ICD-10 codes C15–C26.

Exposure to ionizing radiation, including internal contamination from radionuclides such as uranium and radon, has been associated with a range of stochastic health effects, including carcinogenesis, genetic damage, and other somatic disorders [3,4]. Cancer incidence and mortality are considered important epidemiological indicators for assessing the long-term biological effects of chronic low-dose radiation exposure [5]. Available epidemiological evidence suggests that prolonged exposure to low levels of ionizing radiation may contribute to an elevated cancer risk at the population level, although the magnitude of this effect remains difficult to quantify under environmental exposure conditions [6].

Cancer incidence is also regarded as an important ecological indicator reflecting the influence of environmental carcinogens on human health. The etiology of malignant neoplasms is multifactorial and involves anthropogenic pollution, radiation exposure, occupational hazards, oncogenic viruses, and lifestyle-related factors such as tobacco use, poor nutrition, and chronic stress [7]. The mutagenic and carcinogenic effects of uranium have been documented in both international and regional studies, indicating that its chemical toxicity and radiological properties may contribute to long-term health risks, particularly in contaminated territories [8].

Digestive tract cancers (ICD-10 C15–C26) represent a major global health burden. According to GLOBOCAN 2020, cancers of the stomach, colon, liver, and pancreas are among the leading causes of cancer-related mortality worldwide [9]. Several studies have suggested that environmental radiation exposure, particularly through ingestion of contaminated drinking water, may be associated with an increased risk of gastrointestinal malignancies [10,11]. In a population-based case–cohort study conducted in Finland, exposure to natural radionuclides in drinking water was evaluated in relation to stomach cancer risk; although no statistically significant association was identified, the study supported the biological plausibility of radiation-related effects in the digestive system [12]. While that study focused primarily on gastric cancer, the potential carcinogenic relevance of radionuclide exposure may extend to the broader gastrointestinal tract.

In addition to international evidence, environmental investigations conducted in the same study region of Northern Kazakhstan have documented groundwater contamination in settlements located near uranium mining and UWSS. Elevated concentrations of radon, total alpha activity, and other radionuclides were detected in several water sources, indicating long-term radioecological hazards in the area [13]. Although the present study does not directly assess individual exposure to drinking water, these findings provide important environmental context for interpreting patterns of digestive tract cancer in the exposed population.

Our previous study, based on cancer registry data from 2001 to 2015, showed that residents living near uranium waste storage sites in the Stepnogorsk area had a higher overall cancer burden, with digestive tract cancers among the leading cancer groups in the exposed population [14]. These earlier findings did not establish a direct causal relationship between chronic low-dose radiation exposure and digestive tract cancers; however, they indicated the need for continued epidemiological monitoring in this region. Updated data covering 2014–2023 are therefore important for assessing whether previously observed patterns persist, change over time, or differ from those in a control population.

Digestive tract cancers were selected as the primary endpoint for the present study for several reasons. First, previous regional cancer registry analyses in the Stepnogorsk area showed that digestive tract cancers were among the leading cancer groups in populations living near uranium waste storage facilities [9]. Second, digestive tract cancers represent a major public health burden and can be analyzed as a defined group using ICD-10 codes C15–C26. Third, potential exposure pathways in uranium-affected territories may include not only external gamma radiation but also internal exposure through contaminated environmental media [13,15,16,17]. Although radionuclide exposure may affect several organ systems, including the hematopoietic and thyroid systems, leukemia, thyroid cancer, and other radiation-associated outcomes were beyond the scope of the present study and require separate analysis.

Digestive tract cancers have a multifactorial etiology, and established risk factors such as diet, smoking, alcohol consumption, and Helicobacter pylori infection may substantially influence cancer risk [5,18]. Therefore, chronic low-dose radiation exposure should not be interpreted as an isolated cause of digestive tract malignancies, but rather as a possible additional environmental factor that may act together with other known risk factors. In this context, the present study does not aim to prove direct causality, but to describe the epidemiological profile of digestive tract cancers in populations living near uranium waste storage sites.

The aim of the present study was to assess the incidence, structure, and relative risk of digestive tract cancers (ICD-10 C15–C26) among populations exposed and not exposed to UWSS in North Kazakhstan over 10 years (2014–2023). The findings are expected to provide a scientific basis for future preventive measures, public health interventions, and environmental health policy in areas affected by uranium-related environmental contamination. 

## 2. Materials and Methods

### 2.1. Study Design and Population

This retrospective population-based registry study used data from the Electronic Register of Cancer Patients of the Republic of Kazakhstan for the period 2014–2023. All records of patients diagnosed with digestive tract cancers (ICD-10 C15–C26) and registered during this period were screened. Patients were classified into exposed and control groups according to their place of permanent residence.

The exposed group included residents of Stepnogorsk city and the nearby settlements of Aksu, Kvartsitka, and Zavodskoy, which are located in proximity to uranium waste storage sites associated with former uranium mining and processing activities. Previous cancer registry analysis showed that digestive tract cancers were among the most frequent malignancies in this population, which supported the selection of digestive tract cancers as the primary endpoint of the present study [14].

The classification of the exposed area was based on geographical proximity to uranium waste storage sites and on previous environmental and radiation–hygienic studies documenting gamma radiation, radon, and radionuclide contamination in environmental media [13,15,16,18].

The control group included residents of Akkol, located approximately 90 km from the uranium waste storage sites and outside the identified zone of technogenic radiation impact. This population was selected as a comparison group because it had broadly similar demographic and socioeconomic characteristics to the exposed population and was located outside the identified uranium-related impact area.

A minimum residence duration of five years was used as an inclusion criterion to improve the reliability of exposure classification and to reduce potential misclassification related to short-term residence or temporary migration.

### 2.2. Data Collection

Individual-level data were extracted from the national cancer registry and included place of residence, ICD-10 code, diagnosis, cancer stage, date of diagnosis, date of registration, date of deregistration, date of death, cause of death, date of birth, age at diagnosis, sex, and duration of residence.

Records were included in the final analysis if they contained the essential variables required for case identification and group classification, including place of residence, ICD-10 code, and date of diagnosis. Records with missing essential variables were excluded from the final analysis. Variables with occasional missing values, such as sex, were analyzed based on available data and were reported where applicable. No imputation of missing data was performed.

### 2.3. Outcomes and Variables

The primary outcome was the incidence of digestive tract cancers in the exposed and control populations. Secondary variables included sex, age at diagnosis, cancer site, stage at diagnosis, and duration of residence.

### 2.4. Statistical Analysis

Incidence rates were calculated per 100,000 person-years. Because individual-level follow-up time was not available, the denominator for each study group was estimated by summing annual population counts for the corresponding settlements over the relevant study period to approximate person-years. For the overall analysis, annual population counts were summed across 2014–2023. For the temporal analysis, counts were summed separately for 2014–2018 and 2019–2023. Incidence rates were calculated as the number of newly diagnosed cases during each period divided by the estimated person-time for the same period, multiplied by 100,000.

Incidence rate = (number of newly diagnosed cases during the period/estimated person-time during the same period) × 100,000.

Relative risks (RRs) with 95% confidence intervals (95% CIs) were calculated for all digestive tract cancers combined (ICD-10 C15–C26) and for the major site-specific localizations. For temporal analysis, the study period was divided into two intervals: 2014–2018 and 2019–2023. Incidence rates and relative risks were compared across these two periods in the exposed and control populations.

All statistical analyses were performed using IBM SPSS Statistics version 23.0 and StatTech version 3.0.9. A two-sided *p*-value < 0.05 was considered statistically significant.

No formal correction for multiple comparisons was applied because site-specific analyses were considered exploratory. Age-standardized incidence rates were not calculated in the present study because the primary objective was to describe the observed burden and structure of digestive tract cancers in the exposed and control populations. However, age distribution and age-group-specific characteristics were compared between groups to support the interpretation of the findings.

Ethical declaration: The data were retrospectively analyzed, and all ethical principles of the Helsinki Declaration have been followed. The Electronic Register of Cancer Patients of the Republic of Kazakhstan provided researchers with data, with personal information such as name, identity card number, or address blinded. The protocol was reviewed and approved by the Ethics Committee, Protocol No. 41, dated 05 November 2025, held at NJSC Astana Medical University, which is a registered and authorized ethics body under the Ministry of Health of the Republic of Kazakhstan.

## 3. Results

### 3.1. General Characteristics of Patients with Digestive Tract Cancers

During the study period from 2014 to 2023, a total of 588 cases of digestive tract cancers (ICD-10 C15–C26) were identified in the study population. Of these, 465 cases (79.1%) were registered in the exposed group and 123 cases (20.9%) in the control group. The baseline demographic and clinical characteristics of the patients are presented in Table 1.

Men accounted for 249 cases (53.5%) in the exposed group and 77 cases (62.6%) in the control group, while women accounted for 215 cases (46.2%) and 45 cases (36.6%), respectively. One case in each group had missing sex information. Although men predominated in both groups, the difference in sex distribution between groups was not statistically significant (*p* > 0.05). The mean age at diagnosis was 65.1 ± 10.6 years in the exposed group and 65.2 ± 10.4 years in the control group, indicating a highly comparable age profile between the two populations. In the exposed group, the majority of patients were aged 51–70 years (59.8%), followed by those aged >70 years (32.0%). A similar pattern was observed in the control group, where 56.1% of patients were aged 51–70 years and 30.9% were older than 70 years. Cases diagnosed before the age of 35 were rare in both groups.

Cancer stage was recorded according to the stage information available in the national cancer registry. In this study, stages I–II were considered earlier stages, while stages III–IV were considered advanced stages; stage IV generally indicates metastatic disease.

Most digestive tract malignancies were diagnosed at advanced stages. In the exposed group, 37.8% of cases were diagnosed at stage III and 20.0% at stage IV, while stage II accounted for 32.3% and stage I for only 5.6%. In the control group, the distribution was similar, with 39.0% of cases diagnosed at stage III, 16.3% at stage IV, 35.8% at stage II, and 3.3% at stage I. No statistically significant intergroup differences were observed in stage distribution.

Residence duration showed a marked predominance of long-term residence in both groups. Residence duration of 10 years or more was recorded in 83.0% of patients in the exposed group and 61.8% of patients in the control group. This difference between groups was statistically significant (*p* < 0.05), suggesting a higher proportion of long-term residents among patients from the exposed area.

### 3.2. Structure of Digestive Tract Cancers by Localization

The distribution of digestive tract cancers by anatomical site showed a similar general pattern in both groups. The distribution of digestive tract cancers by localization is shown in Table 2.

In the exposed population, the most common malignancies were colorectal cancer (C18–C21), accounting for 177 cases (38.1%), followed by stomach cancer (C16) with 137 cases (29.5%) and pancreatic cancer (C25) with 81 cases (17.4%). Esophageal cancer (C15) accounted for 43 cases (9.2%), while liver cancer (C22) represented 19 cases (4.1%). Gallbladder and biliary tract cancers (C23–C24) were relatively rare, with 6 cases (1.3%), and only 1 case (0.2%) was classified as C26.

A comparable pattern was observed in the control group. Colorectal cancer was also the leading site, with 49 cases (39.8%), followed by stomach cancer with 38 cases (30.9%), and pancreatic cancer with 13 cases (10.6%). Esophageal cancer accounted for 11 cases (8.9%), liver cancer for 4 cases (3.3%), and gallbladder/biliary tract cancers for 6 cases (4.9%). One case of small intestine cancer (C17) was identified in the control group.

Thus, in both populations, colorectal and stomach cancers constituted the largest share of digestive tract malignancies. Although the overall structure of localization was broadly comparable, pancreatic cancer represented a relatively larger proportion in the exposed population, whereas gallbladder and biliary tract cancers were proportionally more frequent in the control group.

### 3.3. Incidence Rates and Relative Risks

**Figure 1:**
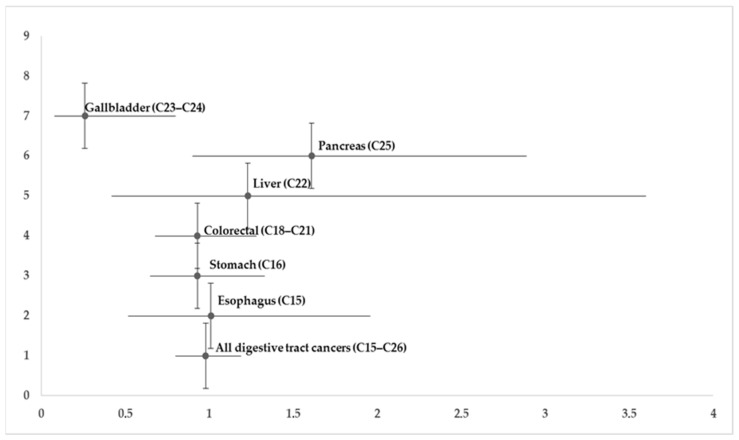
Relative risks (RRs) with 95% confidence intervals (95% CIs) for overall and site-specific digestive tract cancers in the exposed group compared with the control group. The vertical reference line indicates RR = 1.0.

Using population data for 2014–2023, the incidence rate of all digestive tract cancers combined (C15–C26) was 85.27 per 100,000 population in the exposed group and 87.40 per 100,000 population in the control group. The corresponding relative risk (RR) was 0.98 (95% CI: 0.80–1.19), indicating no overall excess risk of digestive tract malignancies in the exposed population when all C15–C26 sites were analyzed together. Relative risks with 95% confidence intervals for overall and site-specific digestive tract cancers are shown in Figure 1.

Site-specific analyses revealed some variation across cancer localizations. For esophageal cancer (C15), the RR was 1.01 (95% CI: 0.52–1.96), indicating no meaningful difference between groups. For stomach cancer (C16), the RR was 0.93 (95% CI: 0.65–1.33), and for colorectal cancer (C18–C21) the RR was also 0.93 (95% CI: 0.68–1.28), again suggesting no significant elevation in the exposed population.

For liver cancer (C22), the RR was 1.23 (95% CI: 0.42–3.60), based on a relatively small number of cases, and therefore should be interpreted cautiously. The highest site-specific point estimate was observed for pancreatic cancer (C25), with an RR of 1.61 (95% CI: 0.90–2.89). Although this did not reach statistical significance, it suggests a potentially elevated burden of pancreatic cancer in the exposed group that may warrant further investigation. In contrast, gallbladder and biliary tract cancers (C23–C24) were less frequent in the exposed group, with an RR of 0.26 (95% CI: 0.08–0.80).

Overall, these results indicate that while the combined burden of digestive tract cancers was similar in the exposed and control groups, variation was observed at the level of specific cancer sites, particularly for pancreatic and biliary tract malignancies.

### 3.4. Temporal Trends in 2014–2023 and 2019–2023 

**Figure 2:**
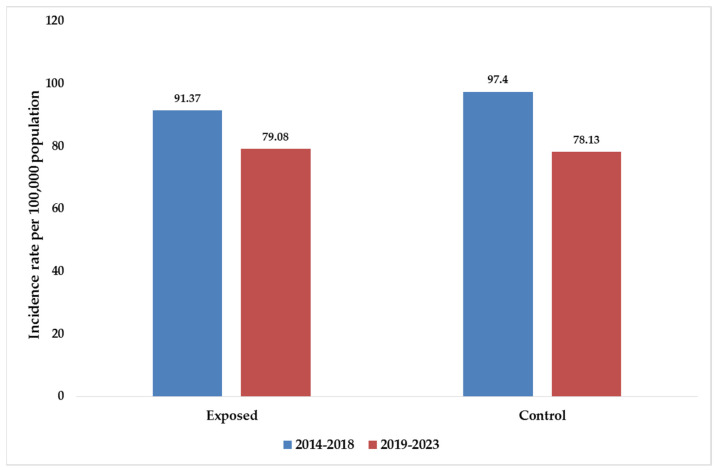
Incidence rates of digestive tract cancers (ICD-10 C15-C26) in the exposed and control groups during 2014–2018 and 2019–2023. Rates are expressed per 100,000 person-years.

When the study period was divided into two five-year intervals, 251 cases (54.0%) of digestive tract cancers were registered in the exposed group during 2014–2023, compared with 214 cases (46.0%) during 2019–2023. In the control group, 66 cases (53.7%) occurred during the first period and 57 cases (46.3%) during the second period, indicating a similar temporal distribution. Incidence rates in the exposed and control populations across the two study periods are shown in Figure 2.

In the exposed population, the incidence rate for all C15–C26 cancers was 91.37 per 100,000 during 2014–2023 and 79.08 per 100,000 during 2019–2023. In the control population, the corresponding incidence rates were 97.40 per 100,000 and 78.13 per 100,000, respectively.

The relative risk for all digestive tract cancers was 0.94 (95% CI: 0.72–1.23) during 2014–2023 and 1.01 (95% CI: 0.76–1.36) during 2019–2023. These findings suggest that the overall incidence of digestive tract cancers remained broadly stable over time and that no statistically significant intergroup difference emerged in either time interval.

## 4. Discussion

The present study provides a population-based assessment of the incidence and structure of digestive tract cancers among populations exposed and not exposed to UWSS in North Kazakhstan over 10 years (2014–2023). The main finding is that no statistically significant increase in the overall incidence of digestive tract cancers (ICD-10 C15–C26) was observed in the exposed group compared with the control group. This result should be interpreted within the limits of a retrospective registry-based design. It indicates that, during the study period, the overall burden of digestive tract cancers was statistically comparable between the exposed and control populations, but it does not exclude the possible contribution of environmental factors to site-specific cancer patterns or individual risk. The absence of a detectable increase in overall cancer incidence may reflect the characteristics of chronic low-dose radiation exposure, where biological effects are typically subtle, cumulative, and may require extended latency periods to manifest at the population level. While the carcinogenicity of ionizing radiation is well established, most epidemiological evidence derives from high-dose exposures, whereas the effects of low-dose environmental exposures remain more difficult to quantify and may not yield clear population-level signals. These effects may be mediated through complex biological mechanisms, including immune modulation and adaptive responses to low-dose radiation exposure [19]. In addition, cancers of the digestive tract are strongly influenced by non-radiation-related risk factors, including diet, Helicobacter pylori infection, alcohol consumption, and smoking, which may obscure or attenuate potential radiation-related associations [20]. For this reason, the observed cancer patterns should not be interpreted as evidence of a direct causal effect of chronic low-dose radiation exposure alone. The findings are better understood as an epidemiological description of cancer incidence in a population living under specific environmental conditions. In the absence of individual data on lifestyle factors, infectious risk factors, and personal radiation dose, the role of chronic low-dose radiation exposure can only be considered as a possible additional factor requiring further investigation.

Importantly, the findings should be interpreted in the context of documented environmental contamination in the study region. Previous studies conducted in Northern Kazakhstan have demonstrated elevated levels of radon and radionuclides in groundwater, particularly in settlements located near uranium mining and UWSS [15,16,18]. In certain locations, radon concentrations in borehole water exceeded recommended limits by an order of magnitude, indicating substantial environmental exposure and potential internal radiation dose through ingestion. Despite this confirmed exposure, the absence of an overall increase in digestive cancer incidence in the present study highlights the complexity of carcinogenesis under conditions of chronic environmental radiation exposure and suggests that any potential effects may be small, organ-specific, or modified by other risk factors.

The results of the present study are consistent with findings from previous epidemiological research. In particular, a population-based case–cohort study from Finland did not demonstrate a statistically significant association between exposure to naturally occurring radionuclides in drinking water, including radon, radium, and uranium, and the risk of stomach cancer, even in populations with relatively high exposure levels [12]. Similarly, earlier ecological studies have yielded inconsistent findings regarding the relationship between environmental radionuclide exposure and gastrointestinal malignancies [17,21]. Together, these findings suggest that low-dose internal exposure to naturally occurring radionuclides may not necessarily translate into a measurable increase in gastrointestinal cancer incidence at the population level. A similar pattern of substantial gastrointestinal cancer burden has also been observed in Kazakhstan for colorectal cancer, where recent national registry data demonstrated persistent mortality, stage-dependent survival differences, and marked regional variation over 2014–2023 [22].

Despite the absence of an overall effect, site-specific analysis revealed some heterogeneity in risk patterns, most notably a higher relative risk for pancreatic cancer in the exposed population. Although this finding did not reach statistical significance, it may indicate a potential localized or organ-specific response to environmental factors and should not be disregarded. This observation is of particular interest in the national context, since a recent population-based study from Kazakhstan demonstrated increasing pancreatic cancer incidence and mortality during 2014–2023, together with very poor survival, with a 5-year survival rate of only 10.9%, and the majority of cases diagnosed at advanced stages [23]. Given the biological characteristics of pancreatic cancer and its sensitivity to metabolic and environmental influences, this observation warrants further investigation in studies incorporating individual exposure assessment and multivariate modeling.

Another important observation is the predominance of advanced-stage diagnoses in both populations. The high proportion of stage III–IV cancers suggests persistent challenges in early detection and access to timely diagnostic services. This interpretation is supported by recent nationwide data from Kazakhstan on other gastrointestinal malignancies. A national study of esophageal cancer reported that more than 94% of cases were diagnosed at stage II–IV, with a marked increase in mortality and disease burden over 2014–2023, highlighting persistent limitations in early detection and timely oncological care [24,25]. This finding underscores the importance of strengthening cancer screening and early detection programs, particularly in regions with potential environmental health risks.

The higher proportion of long-term residents in the exposed group may reflect cumulative exposure to environmental factors over time. However, the lack of a corresponding increase in cancer incidence suggests that prolonged residence alone does not necessarily translate into a measurable risk increase, at least within the timeframe and exposure levels observed in this study.

Temporal analysis did not reveal any significant changes in incidence between the periods 2014–2018 and 2019–2023, nor did it demonstrate divergence between the exposed and control groups over time. This stability further supports the conclusion that no emerging increase in digestive tract cancer incidence associated with environmental exposure was observed during the study period.

Several limitations should be considered. First, the analysis was based on non-standardized incidence rates, which may limit comparability between populations with different age structures. Second, individual-level exposure data, including measured radon concentrations, uranium intake, drinking-water exposure, and cumulative radiation dose, were not available; therefore, exposure classification was based on place of residence. Third, important confounding factors such as diet, smoking, alcohol consumption, occupational exposures, socioeconomic status, and Helicobacter pylori infection were not available in the registry dataset. This limits causal interpretation and may have influenced the observed associations. Fourth, the relatively small number of cases for certain cancer sites, particularly liver, biliary tract, and pancreatic cancers, reduced statistical power for site-specific analyses. Therefore, the results should be interpreted as descriptive epidemiological findings rather than evidence of a direct causal relationship. Future studies should include individual exposure assessment, standardized incidence calculations, and data on major lifestyle and clinical risk factors.

## 5. Conclusions

This study evaluated the incidence, structure, and relative risk of digestive tract cancers among populations exposed and not exposed to UWSS in North Kazakhstan during 2014–2023. A total of 588 cases of digestive tract cancers (ICD-10 C15–C26) were identified, including 465 cases (79.1%) in the exposed group and 123 cases (20.9%) in the control group.

The mean age at diagnosis was similar in both groups: 65.1 ± 10.6 years in the exposed group and 65.2 ± 10.4 years in the control group. No statistically significant differences were observed in sex distribution or cancer stage between the groups. In both populations, most cases were diagnosed at advanced stages, with stage III accounting for 37.8% in the exposed group and 39.0% in the control group, and stage IV accounting for 20.0% and 16.3%, respectively.

The overall incidence rates were comparable between groups: 85.27 per 100,000 in the exposed group and 87.40 per 100,000 in the control group. The relative risk for all digestive tract cancers combined did not indicate a statistically significant increase in the exposed population (RR = 0.98; 95% CI: 0.80–1.19). The structure of digestive tract cancers was also broadly similar, with colorectal and stomach cancers being the leading localizations in both groups. Colorectal cancer accounted for 38.1% of cases in the exposed group and 39.8% in the control group, while stomach cancer accounted for 29.5% and 30.9%, respectively.

Site-specific analysis showed no statistically significant differences for esophageal, stomach, or colorectal cancers. Pancreatic cancer showed a higher, although not statistically significant, relative risk in the exposed population (RR = 1.61; 95% CI: 0.90–2.89), which requires further investigation in larger studies with individual exposure assessment.

Temporal analysis showed a decrease in incidence rates in both populations between 2014–2018 and 2019–2023: from 91.37 to 79.08 per 100,000 in the exposed group and from 97.40 to 78.13 per 100,000 in the control group. The relative risk remained non-significant in both periods.

Overall, the results indicate that the incidence, structure, and temporal trends of digestive tract cancers were statistically comparable between the exposed and control populations during the study period. The findings should be interpreted with caution because the study was based on retrospective registry data, non-standardized incidence rates, and residence-based exposure classification. Individual measurements of radiation dose, radon exposure, uranium intake, lifestyle factors, occupational exposures, and Helicobacter pylori infection were not available.

Future studies should include standardized incidence calculations, individual exposure assessment, and data on major lifestyle, infectious, occupational, and clinical risk factors. Such studies would help researchers to better clarify the possible contribution of uranium-related environmental exposure to site-specific digestive tract cancer risk.

## Figures and Tables

**Table 1:** Baseline characteristics of patients with digestive tract cancers, 2014–2023.

Variable	Exposed (*n* = 465)	Control (*n* = 123)	*p*-Value
Male, *n* (%)	249 (53.5)	77 (62.6)	>0.05
Female, *n* (%)	215 (46.2)	45 (36.6)	>0.05
Mean age, years (±SD)	65.1 ± 10.6	65.2 ± 10.4	>0.05
Age 51–70, *n* (%)	59.8	56.1	>0.05
Age > 70, *n* (%)	32.0	30.9	>0.05
Stage I, *n* (%)	5.6	3.3	>0.05
Stage II, *n* (%)	32.3	35.8	>0.05
Stage III, *n* (%)	37.8	39.0	>0.05
Stage IV, *n* (%)	20.0	16.3	>0.05
Residence ≥ 10 years, *n* (%)	83.0	61.8	<0.05

**Table 2:** Distribution of digestive tract cancers by cancer site (ICD-10 C15-C26).

Cancer Site	Exposed *n* (%)	Control *n* (%)
Colorectal (C18–C21)	177 (38.1)	49 (39.8)
Stomach (C16)	137 (29.5)	38 (30.9)
Pancreas (C25)	81 (17.4)	13 (10.6)
Esophagus (C15)	43 (9.2)	11 (8.9)
Liver (C22)	19 (4.1)	4 (3.3)
Gallbladder and biliary tract (C23–C24)	6 (1.3)	6 (4.9)
Other	1 (0.2)	1 (0.8)

*Note: “Other” includes less frequent digestive tract cancer sites within ICD-10 C15–C26 that were not presented as separate categories because of small case numbers, including C17 and C26.*

## Data Availability

The data used in this study were obtained from the Electronic Register of Cancer Patients of the Republic of Kazakhstan. The data are not publicly available due to ethical and legal restrictions related to personal health information. Data sharing does not apply to this article.

## Abbreviations

The following abbreviations are used in this manuscript:

CI	Confidence interval
GLOBOCAN	Global Cancer Observatory
ICD-10	International Classification of Diseases, 10th Revision
RR	Relative risk
